# Vitamin D Supplementation Impacts Calcium and Phosphorus Metabolism in Piglets Fed a Diet Contaminated with Deoxynivalenol and Challenged with Lipopolysaccharides

**DOI:** 10.3390/toxins15060394

**Published:** 2023-06-13

**Authors:** Béatrice Sauvé, Younes Chorfi, Marie-Pierre Létourneau Montminy, Frédéric Guay

**Affiliations:** 1 Department of Animal Sciences, Laval University, Quebec, QC G1V 0A6, Canada; beatrice.sauve.1@ulaval.ca (B.S.); marie-pierre.letourneau-montminy.1@ulaval.ca (M.-P.L.M.); 2 Department of Veterinary Biomedicine, Montreal University, Saint-Hyacinthe, QC J2S 2M2, Canada; younes.chorfi@umontreal.ca

**Keywords:** piglets, deoxynivalenol, vitamin D, mineralization, calcium, phosphorus, lipopolysaccharides

## Abstract

Using alternative feed ingredients in pig diets can lead to deoxynivalenol (DON) contamination. DON has been shown to induce anorexia, inflammation, and—more recently—alterations in the vitamin D, calcium, and phosphorus metabolisms. Adding vitamin D supplementation in the form of vitamin D_3_ and 25-OH-D_3_ to the feed could modify the effects of DON in piglets. In this study, vitamin D_3_ or 25-OH-D_3_ supplementation was used in a control or DON-contaminated treatment. A repetitive exposure over 21 days to DON in the piglets led to disruptions in the vitamin D, calcium, and phosphorus metabolisms, resulting in a decreased growth performance, increased bone mineralization, and the downregulation of genes related to calcium and to phosphorus intestinal and renal absorption. The DON challenge also decreased blood concentrations of 25-OH-D_3_, 1,25-(OH)_2_-D_3_, and phosphate. The DON contamination likely decreased the piglets’ vitamin D status indirectly by modifying the calcium metabolism response. Vitamin D supplementations did not restore vitamin D status or bone mineralization. After a lipopolysaccharide-induced inflammatory stimulation, feeding a 25-OH-D_3_ supplementation increased 25-OH-D_3_ concentration and 1,25-(OH)_2_-D_3_ regulations during the DON challenge. DON contamination likely induced a Ca afflux by altering the intestinal barrier, which resulted in hypercalcemia and hypovitaminosis D. The vitamin D supplementation could increase the calcitriol production to face the combined LPS and DON challenge.

## 1. Introduction

In the swine industry, feeding accounts for more than 60% of the production cost. Moreover, it has significantly fluctuated over the last few years, making the production hardly profitable [1]. In this context, reducing feeding costs remains a challenge. One option is to diversify raw materials by adding by-products and low graded grain [2]. However, this type of diversification often leads to the use of ingredients contaminated with mycotoxins. The most common mycotoxin detected in swine diets is deoxynivalenol (DON), which is produced by the *Fusarium* fungus [3,4]. This secondary metabolite, part of the trichothecene group, is found in wheat, barley, oats, millet, and corn [5]. It can withstand high temperatures and processing, resulting in its widespread occurrence in animal feed with an occurrence ranging from 96% to 100% [6,7]. The effect of DON on growth performance is well known in pigs [3]. DON contamination is reported to decrease feed intake and leads to reduced growth; in addition, it sometimes leads to complete feed refusal at higher doses (3.0–8.0 mg/kg) [8,9,10], even though the concentration of DON found in feed rarely exceeds the 1000 μg/kg limit [7]. At low doses, DON contamination does not always impact growth performance [11,12]. Pigs are very sensitive to DON as it alters immune function [9], as well as induces oxidative stress [13] and intestinal damage [14]. In addition to these known effects, Le Thanh et al. [8] also observed that DON improved calcium (Ca) retention, as well as reduced Ca and phosphorus (P) excretion in pigs, suggesting a possible DON action on the phosphocalcic metabolism.

**Figure 1:**
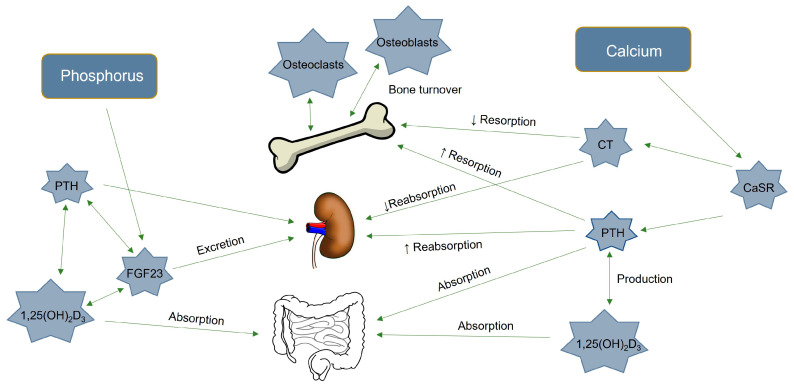
The calcium and phosphorus metabolism, and the main hormones implicated in the phosphocalcic regulations. CaSR: calcium sensing receptor, PTH: parathormone, CT: calcitonin, 1,25-OH_2_D_3_: calcitriol, and FGF23: Fibroblast growth factor 23.

Vitamin D is produced by UV light photolytic action on the skin, but it can also enter the body through dietary intake [15]. The resulting vitamin D_3_ is then hydroxylated at the C-25 position to form 25-hydroxyvitamin D_3_ (25-OH-D_3_) in the liver by the action of the enzyme 25-hydroxylase, which is regulated by the CYP2R1 gene. The 25-OH-D_3_ is then hydroxylated at the C-1 position in the kidney to form 1,25-OH_2_-D_3_, which is the active and hormonal form of vitamin D and is also called calcitriol. The latter step is controlled by the enzyme 1α-hydroxylase, which is regulated by the CYP27B1 gene. This active form is responsible for most of the biological actions of vitamin D [16,17]. Calcitriol acts on the intestine to increase Ca and P absorption, and on the bone to further increase bone resorption [18,19]. It also decreases parathyroid hormone (PTH) synthesis by negative feedback control (Figure 1) [20]. Increased 1,25-OH2-D_3_ and PTH also stimulate fibroblast growth factor 23 (FGF23) production, which inhibit the type 2a and 2c sodium-phosphate cotransporters (SLC34 and SLC20) that are responsible, depending on its coreceptor Klotho, for kidney P reabsorption [19,21]. The TRPV5 and TRPV6 channels—found in the kidney and intestine, respectively—are responsible for the apical influx of the active Ca transport, and their regulation follows the serum 1,25-OH_2_-D_3_ concentration [22]. Additionally, PTH, FGF23, Klotho, Ca, and P are regulators of the synthesis and activity of 1,25-OH_2_-D_3_ [15,23]. It was observed that DON contamination occurred only at a daily dose of 10 mg/kg administered (body weight) perorally for 7 days, and that this decreased 25-OH-D_3_ concentration in the blood and 25-hydroxylase activity in the liver of rats [24]. Vitamin D also shows immunoregulatory functions that are capable of reducing the inflammatory response [16,25]. Since DON can also induce inflammation and oxidative stress [3,5], adding vitamin D to piglets’ diet may be protective. Moreover, vitamin D supplementation could prevent growth depression [26] and vitamin D deficiency in piglets [27,28].

DON contamination is known to impact the integrity of the intestinal barrier. It increases the paracellular permeability in intestinal porcine cells IPEC-1 at a 30 μmol/L dose [29], and damages the apical structure of tight junction zona occludens-1 in the mid-jejunum of pigs that are fed a 3.1 mg/kg DON-contaminated feed [30]. DON contamination also alters the immune system’s response, leading to reduced resistance to pathogen-associated molecular patterns such as lipopolysaccharides (LPS) [31], which also act on the intestinal barrier. The LPS produced by Gram-negative bacteria are commonly found in livestock environments or directly in pigs’ intestinal microbiota [32], and they induce a systemic inflammatory response at low doses [33]. Thus, LPS and DON could interact together to further increase intestinal permeability and bacterial translocation [34], leading to an amplified immune response [35].

The aim of this experiment was to investigate the effect of dietary DON and vitamin D_3_ supplementation on growth performance, bone mineral content (BMC), blood parameters (including calcium, phosphate, magnesium, 25-OH-D_3_, and 1,25-OH_2_-D_3_), the gene expressions related to the intestinal absorption and renal reabsorption of Ca and P, as well as vitamin D metabolism. Additionally, the study aimed to evaluate the effect of vitamin D supplementation on the same parameters in piglets that are challenged with LPS and fed with a DON-contaminated diet.

## 2. Results

### 2.1. Growth Performance, Bone Mineralization

It is worth mentioning that the total Ca was higher than expected in the experimental diet after analysis of the feed composition (Table A1), especially in the DON diets. It is possible that a higher dietary supply of Ca influenced the response of DON on the Ca and vitamin D metabolisms. No interaction between the DON contamination and vitamin D supplementation was observed for growth performance or bone mineralization. The ADG, the ADFI, and the final body weight (BW) were lower in the piglets fed DON-contaminated feed (*p* < 0.001; Table 1), while the G:F was not affected. The BMC was reduced for the piglets fed DON-contaminated feed compared to the CON (*p* < 0.001), while the percentage for the BMC/kg of BW was higher for piglets fed the DON-contaminated feed compared to the CON (*p* < 0.001). Vitamin D supplementation did not impact the growth performance or bone mineralization.

### 2.2. Blood Parameters

**Figure 2:**
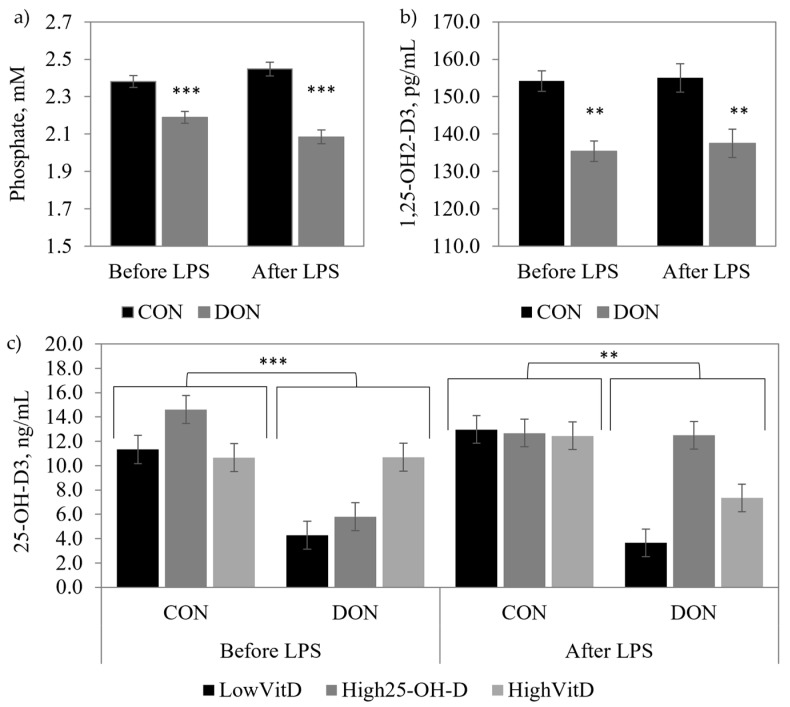
Effect of DON contamination, vitamin D supplementation, and LPS stimulation on phosphate, 1,25-OH_2_-D_3,_ and 25-OH-D_3_ concentrations in the blood of piglets after a repetitive exposure over 21 days. (**a**) Plasma phosphate before and after LPS stimulation; (**b**) serum 1,25-OH_2_-D_3_ before and after LPS stimulation; and (**c**) interaction of DON × VitD for plasma 25-OH-D_3_ before and after LPS stimulation. DON contamination was obtained from naturally contaminated wheat (4.9 mg/kg). The vitamin D supplementations were low vitamin D_3_ (LowVitD, 200 UI vitamin D_3_/kg), high vitamin D_3_ (HighVitD, 2200 UI vitamin D_3_/kg), or high 25-hydroxyvitamin D_3_ (High25-OH-D_3_, 2000 UI in the form of 25(OH)D_3_/kg [0.05 mg/kg]). One piglet per pen received intraperitoneal LPS injection (20 µg/kg BW (20 µg/kg). CON: control, DON: deoxynivalenol, LPS: lipopolysaccharide, and VitD: vitamin D_3_, 25-OH-D_3_: 25-hydroxyl-Vitamin D_3_. The data are reported as the mean ± SEM. ** *p* < 0.05; *** *p* < 0.01.

Piglets unchallenged with LPS and fed DON-contaminated diets had higher serum DON and DOM-1 concentrations than the CON piglets (*p* < 0.001; Table 2), and High25-OH-D_3_ supplementation tended to reduce DOM-1 concentration (DON × VitD; *p* = 0.09). Serum Ca and Mg were not modified by the DON contamination or vitamin D supplementation in piglets that were or were not injected with LPS. DON contamination decreased the plasma concentration of 25-OH-D_3_ (*p* < 0.05; Figure 2b), phosphate (*p* < 0.01; Figure 2a), and the serum concentration of 1,25-(OH)_2_-D_3_ (*p* < 0.01; Figure 2c). The same results were observed in piglets challenged with the LPS for 25-OH-D_3_ (*p* < 0.05), phosphate (*p* < 0.001), and 1,25-(OH)_2_-D_3_ (*p* < 0.05). The High25-OH-D_3_ treatment tended to prevent a decrease of 25-OH-D_3_ (DON × VitD; *p* = 0.09) in the piglets that were fed the DON diet and injected with LPS. There was no interaction between DON contamination and vitamin D supplementation for the serum/plasma concentrations of DON, 1,25-(OH)_2_-D_3_, P, Ca, and Mg in piglets that were or were not injected with LPS (Table 2 and Figure 2).

### 2.3. Gene Expression

**Figure 3:**
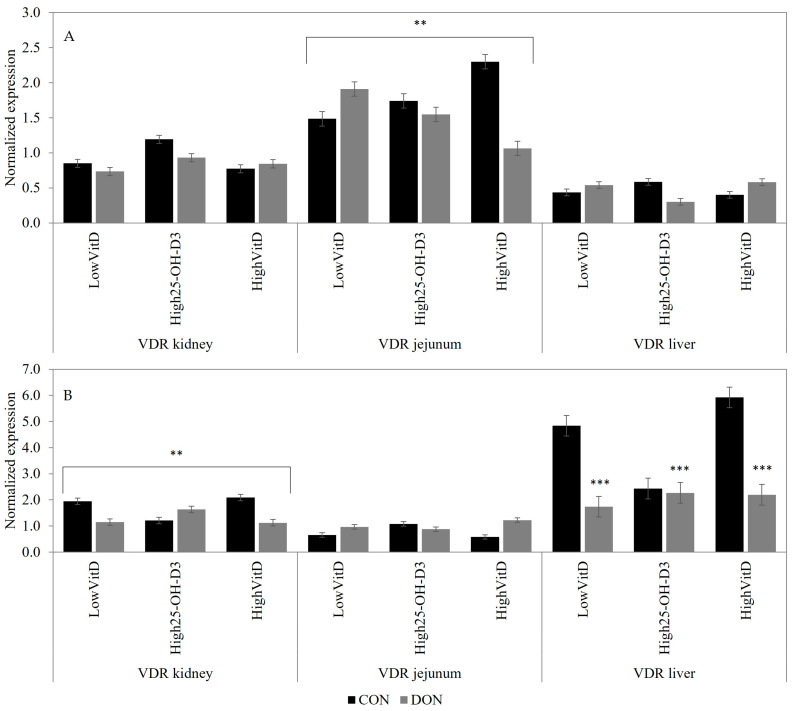
Effect of DON contamination, vitamin D supplementation, and LPS stimulation on the vitamin D receptor (VDR) gene expression in the kidney, jejunum, and liver after a repetitive exposure over 21 days. (**A**) VDR gene expression before LPS stimulation; VDR in jejunum showed an interaction between DON × VitD. (**B**) VDR gene expression after LPS stimulation; VDR in kidney showed an interaction between DON × VitD. VDR in liver was analyzed with a Poisson adjustment. DON contamination was obtained from naturally contaminated wheat (4.9 mg/kg). The vitamin D supplementations were low vitamin D_3_ (LowVitD, 200 UI vitamin D_3_/kg), high vitamin D_3_ (HighVitD, 2200 UI vitamin D_3_/kg), or high 25-hydroxyvitamin D_3_ (High25-OH-D_3_, 2000 UI in the form of 25(OH)D_3_/kg [0.05 mg/kg]). One piglet per pen received intraperitoneal LPS injection (20 µg/kg BW (20 µg/kg). CON: control, DON: deoxynivalenol, LPS: lipopolysaccharide, and VitD: vitamin D_3_, 25-OH-D_3_: 25-hydroxyl-Vitamin D_3_. The data are reported as the mean ± SEM. ** *p* < 0.05; *** *p* < 0.01.

In piglets not injected with LPS, the DON contamination decreased the jejunal VDR gene expression in piglets with HighVitD and High25-OH-D_3_ supplementations, but it increased LowVitD (DON × VitD; Figure 3A; *p* < 0.05). Piglets injected with LPS and that were receiving DON-contaminated diets had lower VDR (*p* < 0.01; Figure 3B) gene expression in the liver compared to the CON piglets. Additionally, DON contamination decreased the renal VDR gene expression in piglets injected with LPS and that were receiving LowVitD and HighVitD. Meanwhile, in piglets fed High25-OH-D_3_, the opposite results were observed (DON × VitD; Figure 3B; *p* < 0.05).

**Figure 4:**
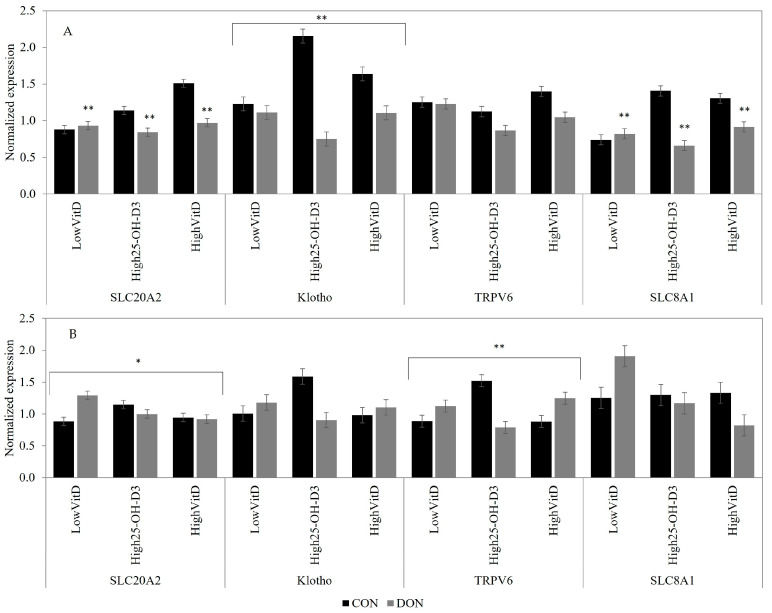
Effect of DON contamination, vitamin D supplementation, and LPS stimulation on gene expression in the jejunum after a repetitive exposure over 21 days. (**A**) Gene expression before LPS stimulation where Klotho showed an interaction between DON × VitD. (**B**) Gene expression after LPS stimulation where the SLC20A2 and TRPV6 showed an interaction between DON × VitD. SLC8A1 was analyzed with a Poisson adjustment. DON contamination was obtained from naturally contaminated wheat (4.9 mg/kg). The vitamin D supplementations were low vitamin D_3_ (LowVitD, 200 UI vitamin D_3_/kg), high vitamin D_3_ (HighVitD, 2200 UI vitamin D_3_/kg), or high 25-hydroxyvitamin D_3_ (High25-OH-D_3_, 2000 UI in the form of 25(OH)D_3_/kg [0.05 mg/kg]). One piglet per pen received intraperitoneal LPS injection (20 µg/kg BW (20 µg/kg). CON: control, DON: deoxynivalenol, LPS: lipopolysaccharide, VitD: vitamin D_3_, 25-OH-D_3_: 25-hydroxyl-Vitamin D_3_., SLC20A2: sodium-dependent phosphate transporter 2, Klotho: fibroblast growth factor 23 coreceptor, SLC8A: sodium/calcium exchanger 1, and TRPV6: transient receptor potential vanilloid 6. The data are reported as the mean ± SEM. * *p* < 0.10; ** *p* < 0.05.

In the jejunum tissue, the DON contamination downregulated the SLC8A1 (*p* < 0.05) and SLC20A2 (*p* = 0.05) gene expression compared to the CON group when the piglets did not receive LPS injection (Figure 4A). In unchallenged LPS piglets, the DON contamination decreased jejunal Klotho gene expression, but the reduction was more pronounced in piglets fed with HighVitD_3_ and High25-OH-D_3_ supplementation when compared to LowVitD_3_ (DON × VitD; Figure 4A; *p* < 0.05). In piglets receiving LPS injection, the DON contamination tended to increase the jejunal SLC20A2 gene expression in piglets fed LowVitD_3_ diets (DON × VitD; Figure 4B; *p* = 0.10). In piglets receiving LPS injection, the High25-OH-D_3_ supplementation in CON diets also increased the jejunal TRPV6 gene expression compared to LowVitD_3_ and HighVitD_3_, but in piglets fed DON-contaminated feed, the opposite result was observed (DON × VitD; Figure 4B; *p* = 0.05).

**Figure 5:**
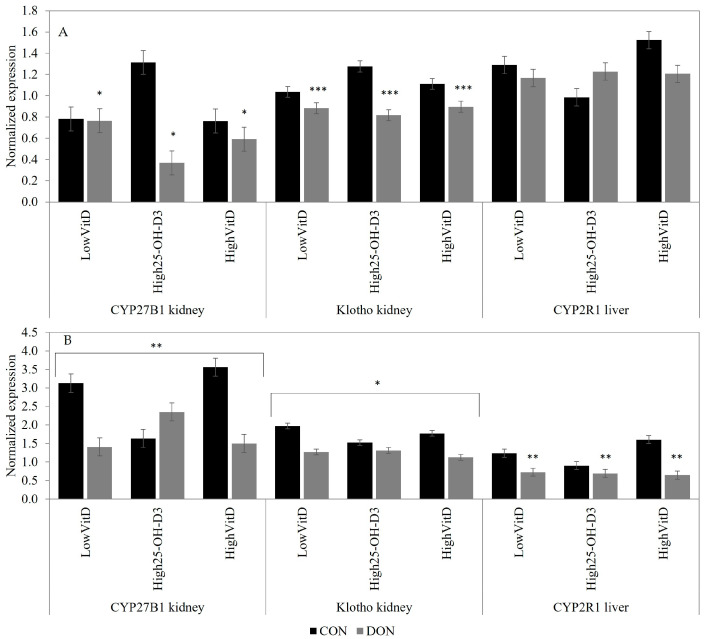
Effect of DON contamination, vitamin D supplementation, and LPS stimulation on the P- and vitamin-D-related gene expression in the kidney and liver after a repetitive exposure over 21 days. (**A**) Gene expression before LPS stimulation. (**B**) Gene expression after LPS stimulation where the renal CYP27B1 and Klotho expressions showed an interaction between DON × VitD. CYP27B1 was analyzed with a Poisson adjustment. The DON contamination was obtained from naturally contaminated wheat (4.9 mg/kg). The vitamin D supplementations were low vitamin D_3_ (LowVitD, 200 UI vitamin D_3_/kg), high vitamin D_3_ (HighVitD, 2200 UI vitamin D_3_/kg), or high 25-hydroxyvitamin D_3_ (High25-OH-D_3_, 2000 UI in the form of 25(OH)D_3_/kg [0.05 mg/kg]). One piglet per pen received intraperitoneal LPS injection (20 µg/kg BW (20 µg/kg). CON: control, DON: deoxynivalenol, LPS: lipopolysaccharide, VitD: vitamin D_3_, 25-OH-D_3_: 25-hydroxyl-Vitamin D_3_, CYP27B1: 1α-Hydroxylase, Klotho: fibroblast growth factor 23 coreceptor, and CYP2R1: 25-Hydroxylase. The data are reported as mean ± SEM. * *p* < 0.10; ** *p* < 0.05; *** *p* < 0.01.

In the liver, only CYP2R1 was evaluated, and its expression was reduced by the DON contamination in piglets injected with LPS (*p* < 0.05; Figure 5A). In the kidney, the DON contamination tended to downregulate the CYP27B1 gene expression in piglets not receiving LPS injection (*p* = 0.10; Figure 5A). In piglets stimulated with an LPS injection, the CYB27B1 gene expression was higher in LowVitD_3_ and HighVitD_3_ than HighVit25-OH-D_3_ in the CON diet, but in the DON-contaminated diets, the opposite result was observed (DON × VitD; Figure 5B; *p* = 0.04). DON contamination reduced the renal Klotho gene expression in piglets not receiving LPS injection (*p* < 0.01; Figure 5A). Klotho gene expression was also downregulated by the DON contamination in piglets injected with LPS, but this effect tended to be reduced in piglets fed a High25-OH-D_3_-supplemented diet (DON × VitD; Figure 5B; *p* = 0.08).

**Figure 6:**
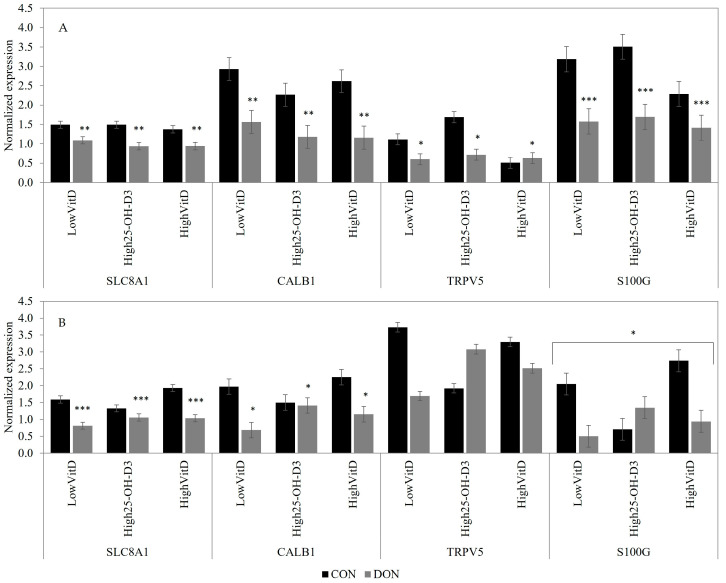
Effect of DON contamination, vitamin D supplementation, and LPS stimulation on Ca-related gene expression in the kidney after a repetitive exposure over 21 days. (**A**) Gene expression before LPS stimulation. (**B**) Gene expression after LPS stimulation where S100G tended to show an interaction between DON × VitD. CALB1 and S100G were analyzed with a Poisson adjustment. DON contamination was obtained from naturally contaminated wheat (4.9 mg/kg). The vitamin D supplementations were low vitamin D_3_ (LowVitD, 200 UI vitamin D_3_/kg), high vitamin D_3_ (HighVitD, 2200 UI vitamin D_3_/kg), or high 25-hydroxyvitamin D_3_ (High25-OH-D_3_, 2000 UI in the form of 25(OH)D_3_/kg [0.05 mg/kg]). One piglet per pen received intraperitoneal LPS injection (20 µg/kg BW (20 µg/kg). CON: control, DON: deoxynivalenol, LPS: lipopolysaccharide, VitD: vitamin D_3_, 25-OH-D_3_: 25-hydroxyl-Vitamin D_3_, SLC8A1: sodium/calcium exchanger 1, CALB-1: Calbindin D28K, TRPV5: transient receptor potential vanilloid 5, and S100G: Calbindin D9K. The data are reported as the mean ± SEM. * *p* < 0.10; ** *p* < 0.05; *** *p* < 0.01.

In piglets not stimulated by LPS, the DON contamination downregulated the SLC8A1 (*p* < 0.05), CALB1 (*p* < 0.05), S100G (*p* < 0.01), and TRPV5 (*p* = 0.06) gene expressions in the kidney (Figure 6A). When stimulating the LPS condition, piglets fed DON-contaminated feed had lower SLC8A1 (*p* < 0.01) and tended to have lower CALB1 (*p* = 0.10) gene expression when compared to the CON group (Figure 6B). Piglets receiving LowVitD_3_ and HighVitD_3_ supplementation tended to have a higher S100G gene expression than High25-OH-D_3_ in piglets fed a CON diet, but an opposite effect was observed in piglets fed a DON-contaminated diet (DON × VitD; Figure 6B; *p* = 0.10). Vitamin D supplementation did not impact the expression of Ca, *p*, and vitamin-D-related genes in the kidney.

## 3. Discussion

No effect of vitamin D supplementation alone was observed in this study. However, vitamin D supplementation sometimes modified the response to DON in piglets that were or were not challenged with LPS.

### 3.1. Impact of DON Contamination and Vitamin D Supplementation

In this study, DON was the only mycotoxin present at a significant amount (DON, 4.9 mg/kg from naturally contaminated wheat), while other mycotoxins such as Aflatoxins, Zearalenone, Fuminosine, Ochratoxin, and T-2 were present in negligible amounts (<1.0 ppb, <0.03 ppm, <0.1 ppm, <0.003 ppm, and <0.06 ppm, respectively). The DON-contaminated diet reduced ADFI and ADG by 36% without affecting feed efficiency. These findings are consistent with previous studies, which have shown that DON contamination induces an anorexic effect in pigs [10,36,37], which can be explained by several mechanisms and thus reduces the gains due to the reduction in energy and protein intake. One of the mechanisms that induce anorexia is linked to the neurotoxic effect of DON on plasma serotonin (5-HT) secretion, which is involved in emesis, nausea, and appetite control [38]. Recently, Wang et al. [39] observed an increased 5-HT secretion in all studied regions of the piglet brain when they were fed a DON-contaminated feed at a concentration of 2.2 mg/kg for 60 days. Generally, a 5-HT increase is partly regulated by the Ca-sensing receptor (CaSR) in the parathyroid gland [40], which is also a Ca and P metabolism regulator.

This link with Ca and P homeostasis and DON may explain the increased bone mineralization per BW observed in the DON treatment groups. In addition, DON contamination clearly reduces the concentrations of 25-OH-D_3_ and 1,25-OH_2_-D_3_, phosphate, and the three other metabolites associated with Ca and P metabolism. For example, 1,25-OH_2_-D_3_ regulates bone remodeling proteins, which maintain the balance between degradation by osteoclasts and the formation by osteoblasts in the bone matrix [41]. In fact, calcitriol acts to maintain Ca homeostasis by stimulating bone resorption and osteoclastogenesis. Osteoblasts increase the release of the receptor activator of nuclear factor kappaB ligand (RANKL), which increases the production and action of osteoclasts [42]. A reduced calcitriol level would decrease the osteoclast production and the bone resorption process. Results from the gene expression in the kidney support a DON effect on Ca metabolism, which is consistent with the BMC results. The CALB1 gene responsible for Ca renal reabsorption [43]—the S100G gene expression that carries out Ca transport [44]—is SCL8A1, and it is responsible for Ca entry in the cell [45]. TRPV5 is responsible for the Ca reabsorption from renal tubules [43] that were all decreased by DON contamination. The jejunal SLC8A1 gene was also downregulated by DON contamination. However, the plasma Ca concentration was not modified by DON contamination. Contrary to our results, Le Thanh et al. [8] observed an increased Ca retention and digestibility, as well as a reduced Ca excretion when piglets weighing 6 kg received a diet contaminated with approximately 4 mg/kg of DON. This increase in Ca and P absorption could be explained by an increase in transcellular and paracellular passages. DON generally decreases the electrical resistance (TEER) of the intestinal barrier and therefore increases its permeability, in addition to increasing the paracellular passage [46]. Le Thanh et al. [8] measured the Ca balance from 9–14 days after beginning the consumption of a DON-contaminated diet in comparison to 21 days in our experiment. The increased permeability of the gut membrane by DON may have induced a high entry of Ca, causing hypercalcemia that was compensated by calcitonin regulations, thus leading to a downregulation of the genes related to the Ca renal and intestinal absorptions observed in the current study.

In the case of P-related genes, the Klotho gene expression in the kidney and jejunum, the coreceptor to FGF23, was decreased by DON contamination. FGF23 is a hypophosphatemic hormone that modulates vitamin D bioactivation [19] and inhibits PTH production in the parathyroid glands [47]. The Klotho expression decrease could be due to the low vitamin D levels induced by DON as the high vitamin D status can upregulate Klotho expression [48]. Jejunal SLC20A2 gene expression, which is responsible for the intestinal absorption of P [49], was decreased by DON contamination. It was previously observed that the P urinary excretion was reduced by DON contamination, but the P digestibility was not modified [8].

In theory, vitamin D supplementation, especially in the bioactive form 25-OH-D_3_, is expected to modify the born turnover process and increase the vitamin D status in CON pigs [42]. In fact, 25-OH-D_3_ is hydroxylated directly into calcitriol and is more efficiently absorbed, whereas vitamin D_3_ needs to be transformed into 25-OH-D_3_ in the liver beforehand [16,50]. However, there was no effect of vitamin D supplementation on the bone mineral content, regardless of its form. The CON treatment met the industry recommendations for vitamin D for feed, but the Ca levels were higher than expected in the present study, which could partly explain the absence of a significant effect from vitamin D_3_ and 25-OH-D_3_ supplementation on the BMC and blood concentrations of 25-OH-D_3_ and 1,25-OH_2_-D_3_. Other authors have also observed no effect in the two forms of vitamin D supplementation on bone mineralization [51,52], while some have shown an effect on serum 25-OH-D_3_ [53] in pigs. In the jejunum, vitamin D supplementation increased the VDR gene expression, which should increase the paracellular transport of vitamin D, P, and Ca [54], but it did not impact blood parameters. In human subjects, an increased Ca intake with a high vitamin D intake caused a decrease in serum 25-OH-D_3_ [55]. In the DON-contaminated pigs, a decrease in plasma 25-OH-D_3_ and serum calcitriol concentrations was observed, and adding vitamin D_3_ or 25-OH-D_3_ to the diet did not change this result. The DON contamination tended to decrease CYP27B1 gene expression in the kidney, which should have been upregulated in response to decreased serum calcitriol [16]. Vitamin D, through the bioactive calcitriol, is known to increase Ca and P intestinal absorption and Ca renal reabsorption [44,56]. Although the apparent total tract digestibility of Ca and P was not measured, none of the evaluated genes related to Ca and P absorption and reabsorption were modified by the vitamin D supplementation. This suggests that vitamin D does not impact the Ca, P, and vitamin D metabolism responses to DON contamination. Thus, DON probably decreased the blood levels of 25-OH-D_3_ and 1,25-OH_2_-D_3_ indirectly as a consequence of the modified Ca metabolism response and increased bone mineralization. DON contamination may also act on the enzyme 24-Hydroxylase, responsible for the calcitriol degradation into 24,25-OH_2_-D_3_ [16], which would partly explain why 25-OH-D_3_ and calcitriol levels remain low even when vitamin D supplementation is added. However, the gene CYP24A1 coding for 24-Hydroxylase was not evaluated.

### 3.2. Impact of DON and Vitamin D Supplement under LPS Challenge

An inflammatory stimulation can occur when piglets are exposed to LPS that is produced by Gram-negative bacteria. Previous studies have shown that an acute LPS challenge with DON exposure increases the response of the innate immune system and systemic circulation [34]. However, the additional stimulation of LPS was not always observed in DON-contaminated pigs. One study found that pigs fed a 3.1 mg/kg DON-contaminated feed and receiving intravenous LPS (7.5 μg/kg of BW) showed no difference in plasma TNF-α and IL-6 concentrations compared to the control group with LPS [31], while another study showed no difference for multiple parameters (aspartate-aminotransferase, albumin, γ-glutamyltransferase) with a similar experimental design [57]. An objective of the current study was thus to evaluate the effect of DON and vitamin D supplements on the Ca, P, and vitamin D metabolisms during an acute inflammatory response induced by LPS.

As described above, DON contamination has induced hypophosphatemia and hypovitaminosis D, as well as reduced the gene expression related to Ca and P reabsorption in the kidney; meanwhile, bone mineralization was increased regardless of whether vitamin D_3_ or 25-OH-D_3_ was added. A plausible explanation is that the increased intestinal barrier permeability caused by DON [46] induced the Ca afflux, leading to hypercalcemia. The pigs responded by reducing their efficiency to reabsorb and resorb bone Ca to reduce calcemia since Ca metabolism is very tightly regulated [58]. Interestingly, piglets stimulated with LPS also had reduced plasma Ca compared to those not receiving a LPS injection (9.6% plasma Ca, P = 0.01, results not shown). This was also observed in dogs stimulated with a 2 μg/kg intravenous LPS injection for 12 h [59]. Holowaychuk et al. [59] observed a decrease in serum ionic Ca (iCa) between 4 h–12 h, and the PTH concentration was increased between 4 h–24 h in response to the hypocalcemia. However, 25-OH-D_3_ concentration started decreasing after 2 h following the LPS challenge. The hypovitaminosis D was in accordance with the iCa decrease in this study, which partly explains the hypocalcemia caused by LPS stimulation [59]. In 12–14 week-old pigs stimulated with a 10 μg/kg intravenous LPS injection, Carlstedt et al. [60] also observed a marked decrease (−17.4%) in iCa within 2 h after LPS injection. Additionally, DON contamination reduced the expression of some genes related to Ca absorption in the jejunum (SLC8A1) and kidney (TRPV5), but—following LPS stimulation—they did not differ. This may be related to the induced hypocalcemia.

Therefore, the LPS challenge may have shifted the systemic response from the hypercalcemia caused by DON to a hypocalcemia response induced by LPS. Additionally, neither vitamin D_3_ nor 25-OH-D_3_ supplementation increased the low vitamin D status caused by the DON challenge without LPS stimulation. However, following the LPS challenge, 25-OH-D_3_ supplementation tended to increase the 25-OH-D_3_ concentration in piglets fed a DON-contaminated diet. Moreover, following the LPS challenge, the response of CYP27B1 and VDR in the kidney to DON was significant but depended on vitamin D supplementation. The 25-OH-D_3_ supplementation increased both renal CYP27B1 and VDR for the piglets receiving DON contamination and LPS challenges, while the opposite response was observed for CON and VitD_3_. CYP27B1 carries out the second hydroxylation that converts 25-OH-D_3_ into calcitriol. The latter participates in the anti-inflammatory response [61], and its biological effects are mediated by its binding to VDR [62,63]. Additionally, the VDR in the liver was decreased by DON during the LPS challenge, suggesting that DON contamination may increase inflammatory conditions through a modulation of the VDR expression. These results showed the beneficial effect of 25-OH-D_3_ to induce a vitamin D response in cases of low Ca status.

## 4. Conclusions

In summary, chronic repetitive exposure over 21 days to DON contamination (4.9 mg/kg) induced anorexia, which decreased the growth performance of piglets. The DON increased bone mineralization per kg of body weight, as well as reduced the circulating 25-OH-D_3_, calcitriol, and phosphate concentrations. As Ca is tightly regulated, the circulating Ca level was maintained, but active intestinal and renal Ca absorption was negatively regulated by DON contamination, thus supporting the hypercalcemia hypothesis. Intestinal P absorption was also negatively regulated by the DON challenge. The concentrations of 25-OH-D_3_ and calcitriol were not increased by their dietary supplementation during the DON challenge, which is associated with the modified Ca metabolism response. After the LPS-induced inflammatory stimulation, feeding a 25-OH-D_3_ supplementation increased the gene expression related to 1,25-(OH)_2_-D_3_ production and its activity during the DON challenge. Therefore, 25-OH-D_3_ supplementation could increase the production of calcitriol, which has anti-inflammatory properties, to face the combined LPS and DON challenge in pigs.

## 5. Materials and Methods

### 5.1. Animals and Feeding Trial

This experiment was performed at the Centre de Recherche en Sciences Animales de Deschambault (Quebec, Canada), and followed the guidelines of the Canadian Council on Animal Care (2009). The protocol (2018-057) was approved by the Institutional Animal Care Committee. All diets fulfilled the NRC requirements (2012 [64]; Table A1), and the supplementation level of vitamin D_3_ was added according to the values proposed by Isabel et al. [65]. Sixty-six castrated male piglets ([Yorkshire × Landrace] × Duroc) weaned at 21 days of age (6.4 ± 0.89 kg) were distributed in 33 pens (2 piglets/pen) depending on their weight at weaning. They were kept in 3 complete blocks with 6 repetitions and 3 incomplete blocks with 5 repetitions depending on the treatment assigned according to the randomization method [66]. The piglets received one of the following six treatments in a 2 × 3 factorial design: control treatments (CON) and DON-contaminated treatments (DON, 4.9 mg/kg from naturally contaminated wheat, Aflatoxins < 1.0 ppb, Zearalenone < 0.03 ppm, Fuminosin < 0.1 ppm, Ochratoxin < 0.003 ppm, T-2 < 0.06) for six pens each. The mycotoxin profile was determined in a commercial laboratory by LC/MS/MS (Actlabs Agriculture, Ancaster, ON, Canada) to confirm that DON was the predominant mycotoxin and that other mycotoxins were present in negligible concentrations. Those treatments were supplemented with low vitamin D_3_ (LowVitD, 200 UI vitamin D_3_/kg; DSM, Belvidere, NJ, USA) or with high vitamin D_3_ (HighVitD, 2200 UI vitamin D_3_/kg, DSM) or high 25-hydroxyvitamin D_3_ (High25-OH-D_3_, 2000 UI in the form of 25(OH)D_3_/kg [0.05 mg/kg, Hy-D^®^, DSM)]). The CON, DON, and CON+HighVitD were in complete blocks with 6 repetitions. The CON+High25-OH-D_3_, DON+ HighVitD, and DON+ High25-OH-D_3_ were in incomplete blocks with 5 repetitions. Upon their arrival, the piglets were fed with a commercial diet (Agri-Marché, St-Isidore, QC, Canada) for a seven-day adaptation period, and then received the experimental diets for 21 days. Piglets were weighed at the beginning and the end of the trial, and feed intake was evaluated after 7 days and 21 days per pen. One piglet per pen received intraperitoneal LPS injection (20 µg/kg BW, Sigma-Aldrich Canada, Oakville, ON, Canada) to induce an acute inflammatory reaction 3 h prior to euthanasia, which was performed with a non-penetrating captive bolt stunner. Blood samples were taken from each piglet 21 days before they were euthanized and 3 h after the LPS injection. Liver, intestinal mucosa, and kidney tissue were then collected. The bone mineral content (BMC) of each piglet was then evaluated after euthanasia with dual-energy X-ray absorptiometry (DXA; Discovery W, Hologic, MA, USA).

### 5.2. Laboratory Analysis

#### 5.2.1. Blood Analysis

Blood samples were taken from all piglets by jugular venipuncture (BD Canada, Mississauga, ON, Canada). Plasma and serum samples were centrifuged at 2000× *g* at 4 °C for 15 min. Plasma and serum samples were kept frozen at −20 °C until analysis. Serum concentrations of DON, DOM-1 [8], phosphate, calcium, and magnesium (Mg) (BioAssay Systems, Hayward, CA, USA) were evaluated. Concentrations of 25-OH-D_3_ (Signalway Antibody, College Park, MD, USA) and 1,25-OH_2_-D_3_ (BioVendor, Brno, Czech Republic) were evaluated in plasma and serum, respectively, by the sandwich ELISA method.

#### 5.2.2. Gene Expression Analysis

Tissue samples of the liver, jejunum mucosa, and kidney were taken to assess the gene expressions related to the P, Ca, and vitamin D metabolisms. They were snap-frozen in liquid nitrogen directly after collection. The mRNA gene expression was quantified according to the method of Lessard et al. [36]. The tissue samples (50 mg) were then homogenized in 1 mL of TRIzol © (Thermo Fisher Scientific, Carlsbad, CA, USA). Then, 200 μL of chloroform was added and samples were centrifuged for 15 min at 12,000× *g* at 4 °C. The aqueous phase was conducted in a new tube, and 500 μL of isopropanol was added. After centrifugation for 10 min at 12,000× *g* at 4 ℃, the isopropanol was removed from the tubes and 75% ethanol was added for a 5 min centrifugation at 7500× *g* at 4 °C. Fifty μL of DNase free water was added to dilute the pellet. The extracted RNA concentration and integrity were assessed with the NanoDrop 1000 Spectrophotometer (Thermo Fisher Scientific, Wilmington, DE, USA), as well as by nucleic acid electrophoresis with an Agilent 2100 Bioanalyzer instrument (Agilent, Santa Clara, CA, USA). Reverse transcription was performed with the qScript Flex (Qiagen Beverly Inc., Cummings, MA, USA), with a 1 μg/μL mRNA concentration. The relative mRNA abundance of genes known to be involved in the vitamin D, Ca, and P metabolisms was quantified using real-time qPCR analyses. Table 2 provides the complete list of selected genes. The qPCR was performed with 10 μL of PerfeCTa^®^ SYBR^®^ Green FastMix^®^ (Quanta Bioscience Inc., Gaithersburg, MD, USA), 1 μL of cDNA, 1 μL of designed primers (Table A2), and 8 μL of RNAse free water using the Lightcycler 480 (Roche, Basel, Switzerland). The PCR cycling conditions were 10 min at 95 °C, followed by 50 cycles at 10 s at 57 °C or 58 °C for primer annealing depending on the gene, and 20 s at 72 °C for the primer extension. A melting curve step was added at 72 °C for 10 s and 94 °C for 5 measures. A relative standard curve was established by serial dilutions of a cDNA pool to determine the mRNA expression levels.

In the first place, the vitamin D receptor (VDR) gene expression was evaluated in all tissues. In the liver, only the CYP2R1 (25-hydroxylase activates vitamin D_3_ into 25-OH-D_3_ in liver) gene expression was also evaluated as the liver does not participate in most of the important regulations for Ca and P absorption. In the kidney and jejunum mucosa, the SLC20A2 (Na-Pi type III transporter, intestinal and kidney absorption of P), Klotho (FGF23 coreceptor), CALB1 (Calbindin-1, Ca kidney transportation), SLC8A1 (Na^2+^/Ca^2+^ 1 exchanger, transfers Ca to blood circulation), and S100G (Calbindin D9K, Ca transportation) gene expressions were assessed. The TRPV6 (transient receptor potential vanilloid 6, transcellular intestinal Ca absorption) gene expression was evaluated in the jejunum. The CYP27B1 (1α-hydroxylase, hydroxylation of 25-OH-D_3_ into 1-25-(OH)_2_-D_3_) and TRPV5 (transient receptor potential vanilloid 5, entry channel of Ca into kidney) gene expressions were evaluated in the kidney. The gene expressions were then normalized with three housekeeping genes, GAPDH, β-Actin, and HPRT.

### 5.3. Statistical Analyses

Growth performance including the average daily feed intake (ADFI), average daily gain (ADG), and the gain-to-feed ratio (G:F) were analyzed in a factorial 2 × 3 design. DON contamination and Vitamin D supplement were set as the fixed effects, and a pen of 2 piglets as the experimental unit with a mixed ANOVA were used on the Minitab software (Minitab20, LLC, State College, PA, USA). For plasma and serum concentrations, and the gene expressions in tissue, a 2 × 3 (DON contamination × Vitamin D supplement as fixed effects) factorial design was used to assess the effects of DON and vitamin D, as well as their interactions before and after LPS injection with a piglet as the experimental unit. Those data were evaluated using a Glimmix procedure on SAS (SAS studio 2021, SAS Inst. Inc. Cary, NC, USA). A *p*-value that was less than 0.05 indicated a significant difference, whereas a *p*-value less than 0.10 indicated a statistical trend.

## Figures and Tables

**Table 1:** Impact of DON contamination supplementation on growth performances and bone mineralization after a repetitive exposure over 21 days ^1^.

	Deoxynivalenol		Vitamin D					*p* Value	
Parameters	CON	DON	Low VitD	High25-OH-D_3_	High VitD	SEM	SD	DON	VitD
ADG, g/d	555.4	358.1	447.2	459.1	466.4	19.4	116.6	0.001	0.66
ADFI, g/d	759.8	493.5	611.0	619.6	649.3	29.3	156.3	0.001	0.51
BMC/BW (%)	1.28	1.41	1.34	1.35	1.34	0.02	0.10	0.001	0.93
BMC, g	253.5	220.3	234.1	234.9	241.4	10.1	40.3	0.001	0.71
G:F	0.73	0.73	0.73	0.74	0.72	0.01	0.05	0.77	0.29
Initial body weight, kg	7.79	7.97	7.96	7.69	7.98	0.30	1.09	0.43	0.50
Final body weight, kg	19.8	15.7	17.6	17.6	18.0	0.61	3.26	0.001	0.72

^1^ Value are least square means. CON: Control diet; DON: deoxynivalenol; BW: body weight; BMC: whole-body bone mineral content; ADFI: average daily feed intake; ADG: average daily gain; and G:F: gain-to-feed ratio.

**Table 2:** Effect of DON contamination, vitamin D supplementation, and LPS stimulation on blood parameters after a repetitive exposure over 21 days ^1^.

Item		DON, ng/mL		DOM-1, ng/mL		Calcium, mM		Magnesium, μM	
Treatments									
Deoxynivalenol	Vitamin D	Before LPS	After LPS	Before LPS	After LPS	Before LPS	After LPS	Before LPS	After LPS
CON	LowVitD	0.21	1.50	0.00	0.00	3.02	2.66	559.53	594.30
CON	High25-OH-D_3_	2.31	0.59	0.00	0.00	2.91	2.83	569.41	679.71
CON	HighVitD	0.90	0.63	0.00	0.00	2.99	2.68	660.50	662.89
DON	LowVitD	23.55	18.31	4.68	4.94	2.76	2.64	521.11	630.23
DON	High25-OH-D_3_	23.16	19.07	3.33	4.09	2.94	2.64	589.56	504.17
DON	HighVitD	30.62	20.28	4.24	5.27	2.90	2.42	601.80	613.01
SEM		2.05	1.96	0.23	0.31	0.12	0.17	56.78	60.02
SD		13.42	11.45	2.27	2.47	0.39	0.45	150.8	165.1
Mixed Procedure		*n* = 32	*n* = 32	*n* = 33	*n* = 33	*n* = 33	*n* = 33	*n* = 33	*n* = 33
DON		<0.001	<0.001	<0.001	<0.001	NS	NS	NS	NS
VitD		NS	NS	0.09	NS	NS	NS	NS	NS
DON × VitD		NS	NS	0.09	NS	NS	NS	NS	NS

^1^ Values are least square means. CON: control, DON: deoxynivalenol, LPS: lipopolysaccharide, NS; Not-Significant, VitD: vitamin D_3_, 25-OH-D_3_: 25-hydroxyl-Vitamin D_3_, SEM: standard error means, and SD: standard deviation.

## Data Availability

The datasets generated during the current study are available from the corresponding authors upon request.

## Appendix A

**Table A1:** Composition of the experimental diets.

	Control ^a^	Control	Control	DON	DON	DON
		+HighVitD	+High25OHD		+HighVitD	+High25OHD
Ingredient (%)						
Control wheat	50.00	50.00	50.00			
Contaminated wheat				50.00	50.00	50.00
Corn	14.06	14.06	14.06	14.06	14.06	14.06
Soybean meal	20.29	20.29	20.29	20.29	20.29	20.29
Hamlet Soy Protein (HP300)	3.03	3.03	3.03	3.03	3.03	3.03
Choice white fat	2.05	2.05	2.05	2.05	2.05	2.05
Whey powder	7.00	7.00	7.00	7.00	7.00	7.00
Monocalcium phosphate	0.88	0.88	0.88	0.88	0.88	0.88
Limestone	1.16	1.16	1.16	1.16	1.16	1.16
Vitamin and mineral premix ^y,z^	0.50	0.50	0.50	0.50	0.50	0.50
Salt	0.30	0.30	0.30	0.30	0.30	0.30
Lysine-HCl	0.45	0.45	0.45	0.45	0.45	0.45
DL-Methionine	0.13	0.13	0.13	0.13	0.13	0.13
L-Threonine	0.15	0.15	0.15	0.15	0.15	0.15
Calculated composition (%)						
Calcium	0.85	0.85	0.85	0.85	0.85	0.85
Phosphorus digestible	0.42	0.42	0.42	0.42	0.42	0.42
Analyzed composition						
Calcium, %	0.94	0.84	0.94	1.05	1.20	1.27
Phosphorus, %	0.65	0.58	0.64	0.60	0.76	0.61
Vitamin D, IU/kg	226	1802	209	170	2444	232
25-OH-D_3_	---	---	2156	---	---	2864
Deoxynivalenol, mg/kg	0.11	0.10	0.18	5.16	4.69	4.98

^a^ Control diet (0.11 mg/kg DON), Control+HighVitD diet (0.10 mg/kg DON), Control+High25-OH-D_3_ diet (0.18 mg/kg DON), DON-contaminated diet (5.16 mg/kg DON), DON+HighVitD diet (4.69 mg/kg DON), DON+High25-OH-D_3_ diet (4.98 mg/kg DON). ^z^ Provided per kilogram of diet: vitamin A palmitate 2000 IU; vitamin D_3_ 200 IU; vitamin E acetate 16 IU; menadione sodium bisulphite 3.75 mg; thiamine HCl 1.0 mg; riboflavin 3.5 mg; niacin 30.0 mg; calcium pantothenate 15.0 mg; pyridoxine HCl 7.0 mg; biotin 0.5 mg; choline bitartrate 375 mg; and vitamin B12 25.0 µg. ^y^ Provided per kilogram of diet: Zn (as zinc carbonate) 100 mg; Fe (as ferric citrate) 100 mg; Cu (as cupric carbonate) 25 mg; I (as potassium iodate) 0.28 mg; Mn (as manganous carbonate) 46 mg; and Se (as sodium selenite) 0.30 mg.

**Table A2:** Primer sequences used for qPCR.

Gene	Primer Sequence (5′-3′)	Product Size, bp	Genebank Accession No.
Vitamin D			
CYP2R1	(F) TTCATCCCTTCTTGACTCCAAC	201	XM_003480731
	(R) TTTATCTGTCACCTGTCACCAC		
VDR	(F) AGGCTTCTTCAGACGGAGCAT	143	NM_001097414.1
	(R) ACTCCTTCATCATGCCGATGT		
CYP27B1	(F) TGGGCTCTCTATGAACTCTCTC	157	DQ295065.1
	(R) GTCTTAGCACTTCCTTGACCAC		
Phosphorus			
Klotho	(F) ACGCGGAACATGACGTACAG	121	XM_013989399.1
	(R) CCTGCAAGGCGATGGAGAT		
SLC20A2	(F) GTGCACCTGCTCTTCCACTTC	106	XM_005657658.1
	(R) ACAAAGCTACCAGAGGACCAATG		
Calcium			
S100G	(F) GAAGGAGGAGCTGAAGCAACTG	139	NM_214140.2
	(R) CACTAACACCTGGAATTCTTCAAAAC		
CALB-1	(F) TGGATCAGTATGGGCAAAGAGA	133	NM_001130226.1
	(R) GTCTTCATGAATTCCTCACAGGACTT		
TRPV5	(F) GCTGCGAGTACGTCGCTATGT	86	XM_003484001.2
	(R) CAGAGGGCTGTTTCTCAGAGAGA		
SLC8A1	(F) AATTGCTAGAGCTACTGTGTATTTCG	136	FJ268730.1
	(R) ATTGGGCTTCTTTATGGTTATTTCT		
TRPV6	(F) TGGGTGTCCCAAAGTCCAAG	95	XM_013985575.1
	(R) ACTCCCTCCTCCTCCCAAAT		
Reference			
GAPDH	(F) CCCCAACGTGTCGGTTGT	91	XM_021091114.1
	(R) CTCGGACGCCTGCTTCAC		
β-Actin	(F) CATCACCATCGGCAACGA	128	XM_003357928.4
	(R) GGATGTCGACGTCGCACTT		
HPRT	(F) TTGTGGTAGGCTATGCCCTTGACT	117	NM_001032376
	(R) CTCAACTTGAACTCTCCTCTTAGG		
